# Experiencing a first food allergic reaction: a survey of parent and caregiver perspectives

**DOI:** 10.1186/1710-1492-9-18

**Published:** 2013-05-29

**Authors:** Zainab B Abdurrahman, Monika Kastner, Cory Wurman, Laurie Harada, Laura Bantock, Heather Cruickshank, Susan Waserman

**Affiliations:** 1 Division of Clinical Immunology and Allergy, Department of Medicine, McMaster University, HSC Room 3 V49, 1280 Main Street West, Hamilton, ON, L8S 4 K1, Canada; 2 St. Michael’s Hospital Li Ka Shing Knowledge Institute, University of Toronto, Toronto, ON, Canada; 3 Department of Health Science, UWO, London, ON, Canada; 4 Anaphylaxis Canada, Toronto, ON, Canada; 5 Anaphylaxis Canada, Kamloops, BC, Canada

**Keywords:** Anaphylaxis, Food allergy, Qualitative methods

## Abstract

**Background:**

Insufficient knowledge of food allergy and anaphylaxis has been identified by caregivers as an important barrier to coping, and a potential cause of fear and anxiety, particularly for those with children newly diagnosed with food allergy.

The purpose of the study was to better understand the experiences of caregivers of children with a first allergic reaction to food, and to identify any deficiencies in the information received at diagnosis.

**Methods:**

A mixed-methods study consisting of an online survey administered to the Anaphylaxis Canada online registry (a patient support group database of approximately 10,000 members), and a follow-up qualitative interview with a subset of survey participants. Analysis consisted of frequency analysis (quantitative and qualitative data) and descriptive statistics to calculate proportions and means with standard deviations. Qualitative analyses were guided by the constant comparative method of grounded theory methodology.

**Results:**

Of 293 survey respondents, 208 were eligible to complete the survey (first allergic reaction to food within 12 months of the study), and 184 respondents consented. Identified gaps included education about food allergy, anaphylaxis management, for example, how to use epinephrine auto- injectors, and coping strategies for fear and anxiety. The qualitative follow-up study supported these findings, yielding 3 major themes: 1) lack of provision of information following the episode on the recognition and management of food allergy related allergic reactions, 2) prolonged wait times for an allergist, and 3) significant family anxiety.

**Conclusions:**

The online survey highlighted multiple deficiencies at diagnosis, findings which were supported by the follow up qualitative study. Results will inform the development of educational strategies for patients newly diagnosed with food allergy.

## Background

Regardless of the healthcare setting, management of food allergy and anaphylaxis remains suboptimal [1,2], and the provision of education to primary care providers, patients and their families is inadequate [3-5]. A systematic review of gaps in anaphylaxis management showed that physicians lack knowledge on how to use epinephrine auto-injectors [6], fail to provide training to patients on their use [6-9], and few refer patients to an allergist even from the Emergency Department(ED) [10,11]. From the patient and caregiver perspective, knowledge gaps include recognition and management of anaphylaxis [8,12-14], food avoidance [15,16], food allergen control in the diet and the environment [17], and how to use epinephrine auto-injectors [14,18-20]. Provision of educational materials by primary care, as well as discharge instructions from the ED on allergen avoidance [11,21] and auto-injectors [7] is also lacking. These deficiencies have been associated with fear and anxiety [22] and are important barriers to coping for parents of children at risk. Furthermore, the continuum of care that leads to a confirmed diagnosis is not well understood. The objectives of this study were to understand the experiences of caregivers of children with first-time allergic reactions to food (within 12 months of the study) and to identify any existing deficiencies in the information they received at diagnosis.

## Methods

A mixed methods study consisting of an Internet-based questionnaire (via Survey Monkey) administered in 2010 (Phase 1); and a follow-up qualitative study conducted in 2011 using telephone interviews and open-ended questionnaires with a subset of survey participants (Phase 2). The study was approved by the McMaster University Hamilton Health Sciences research ethics board. The surveys and interviews were conducted in English. Written informed consent was obtained for all participants and confirmed orally for those participating in the telephone interviews.

### Phase 1: survey

Caregivers of children (age ≤ 18 years) with a newly diagnosed food-related allergic reaction within 12 months of the study were recruited using an e-mail invitation to members of Anaphylaxis Canada. Patients and family members become members of Anaphylaxis Canada on a volunteer basis, although they are also referred to the website by pharmacists, nurses, and physicians. The invitation included a brief email description of the study and the URL link to the survey, which was also available through the Anaphylaxis Canada website (http://www.anaphylaxis.ca/). We used the predefined target population criteria and recruitment resources listed above to sample participants as this method was considered the most appropriate for our study design and research questions [23,24]. It was estimated that approximately 300–500 surveys would be returned (using a response rate of 3-5% from a database of 10,000 people). Analyses consisted of frequency analysis (quantitative and qualitative data) and descriptive statistics to calculate proportions and means with standard deviations (SDs). All statistical analyses were carried out using SPSS (Macintosh version 17.0). Open-ended questions were analysed by two independent coders using grounded theory methodology to identify themes.

### Phase 2: qualitative phone interviews and questionnaires

Phase 1 respondents were invited to participate in a follow-up telephone interview, of which 114 survey respondents expressed interest. Ten participants across Canada were randomly selected for the interviews while the remainder were invited to complete a paper-based version featuring identical questions. Two researchers facilitated the sessions using a structured interview guide (Additional file 1) that was pilot tested for clarity with patients from an allergy clinic at McMaster University. Each interview lasted 30–40 minutes and consisted of open-ended questions about participants’ experiences with their child’s first food related reaction. Interviews were audiotaped and transcribed verbatim. Qualitative analyses were guided by the constant comparative method of grounded theory methodology [24]. Grounded theory is a method of systematically analyzing qualitative data using codes and categories allowing researchers to generate theories based on their data. This methodology is very useful in facilitating the understanding of a phenomenon that has not previously been studied (i.e., how parents or caregivers of children with a first episode food allergy perceive their follow-up visit and their expectations of what should happen), and it enables the exploration of the ways in which the “reality” of the follow-up visit is socially constructed [24]. Data was coded from transcripts using a process of open, axial and selecting coding using NVivo 9 software (QSR International, 2011). Two investigators independently developed a coding scheme by identifying, classifying and labeling the primary content patterns and then identified themes.

## Results

### Phase 1: survey

Of 293 people who accessed the survey, 208 participants (71%) were eligible (newly diagnosed) and 184 participants (63%) consented and completed the survey. Seventy-one percent of the respondents were residents of the province of Ontario; the remainder were from 8 other provinces across Canada. The mean age of the participants’ children was 3.5 years (range 1 month to 17 years). The top four food allergens were peanut (46%), tree nuts (17%), milk (13%), and egg (8%).

#### First encounter with a health care professional

At the time of the first food reaction, 51% of participants took their children to the ED, while the remainder went to family physicians (21%), pediatricians (7%), walk-in clinics (5%), allergists (1%), and nurse practitioners (1.3%). Twenty-one participants (11%) did not take their child to any health care professional. Sixty-two percent of respondents were given the diagnosis of food allergy at the first encounter, of which 43% received information about epinephrine auto-injectors. First prescription for an epinephrine auto-injector was provided by ED physicians (35%), family physicians (21%), paediatricians (6%), and walk-in clinics (1%). The remainder of participants (37%) were given a prescription at the appointment with the allergist. EpiPen® was the most commonly recommended auto-injector (88%). Participants received instruction on how to use an auto-injector from allergists (59%), pharmacists (28%), nurses (13%), or ED physicians (8%); while 10% of participants were self-taught.

#### Provision of information and education

At the first reaction, participants received information on allergen avoidance (67%), symptom recognition (51%) and treatment of a reaction (49%). Most information was delivered in the form of reading materials (46%). Nearly one quarter of respondents (23%) did not receive any information at the first encounter. Other take-home resources included referral to allergy organizations (28%), auto-injector websites (21%), auto-injector training devices (46%), and auto-injector video demonstrations (27%). Allergists were cited as the main provider of resources and information on the management of allergy and anaphylaxis. When asked about the desirability of an educational program after a first reaction, 85% of respondents were interested, and suggested topics such as recognizing an allergic reaction, treating an allergic reaction, teaching others about food allergy, reading food labels, and coping with anxiety. Their preferred method of receiving this information was reading materials and online resources.

#### Referrals and allergy testing

The most common specialist referral by ED physicians and family physicians was to an allergist (77%); followed by pediatricians (25%). Of 142 patients who were referred, 41% received a food allergy diagnosis from the allergist, 13% from the pediatrician, and 11% from the family physician. Most participants (89%) were able to see an allergist within six months; 60% were seen within 2 months. Of 124 participants who received allergy testing, most received a skin prick test (93%), followed by food specific serum IgE (28%) and oral food challenge (5%).

#### Experience of the first reaction

Most participants (84%) recalled being anxious to extremely anxious at the time of first diagnosis. At each step of the care received, many participants (62%) felt that they did not receive enough information about food allergy and anaphylaxis management, epinephrine auto-injectors, and coping strategies. As a consequence of their child’s reaction to food, participants avoided eating out at restaurants (85%), restricted their child’s activities with other children (61%), avoided travel (49%), reduced their work hours (13%), or quit their job in response to the diagnosis (11%). Other lifestyle changes included moving their child to another school or daycare, moving to a different neighbourhood or home schooling their child. Many families reported high levels of anxiety regarding the recognition and management of allergic reactions and anaphylaxis, even though the average score for their level of confidence on these topics was above 2.5 on a 4-point scale (0 being no confidence and 4 being very confident).

### Phase 2: qualitative phone interviews

Ten respondents participated in semi-structured phone interviews, and 7 completed a paper questionnaire with identical questions. All participants were parents of children with food allergies. Table 1 shows the characteristics of the 17 participants (mean age of children at the time of the first reaction was 3 years [range 6 months to 10 years]). Almost 60% of these reactions were due to peanuts or tree nuts.

**Table 1:** **Characteristics of qualitative interview participants** (**N** = **17**)

**Participant characteristics**	**N (%)**
**Gender**	
Men	1 (6)
Women	16 (94)
**Mean age** (**years**)	38 (range 30–47)
**Mean age of allergic children** (**years**)	3 (range 0.5-10)
**First health care contact**	
Emergency Department	10 (59)
Family Physician	4 (23)
Other	3 (18)
**Allergen associated with first reaction**	
Tree nuts	6 (35)
Peanuts	4 (24)
Fish	2 (12)

Three major themes were identified: 1) prolonged wait times to see an allergist for definitive diagnosis, 2) lack of information on symptom recognition and management, and 3) significant family anxiety.

#### Theme 1: first encounter with a health care professional

About half of respondents stated that their physicians lacked knowledge on the recognition, diagnosis, and management of food allergy. Only one patient was referred directly from the ED to an allergist, while the remainder was referred to the family physician or pediatrician. Of the 8 respondents who saw their family physician, 6 felt that their doctor was not well informed:

“I had previously been happy with his care but feel that he ’dropped the ball' on this one.”

Most participants (94%) were referred to an allergist, although this did not always occur on the first visit after the food allergic episode. Some parents had to convince their physicians to refer their child to an allergist.

“I don’t think she … actually believed that my daughter had an allergy. She, at first was a bit reluctant to send us and then she said okay, I’ll send you”

#### Theme 2: provision of information

Of 14 participants who indicated lack of provision of information after the first reaction, 76% described having to find information on their own. Although their experiences in the ED varied, they all shared a similar experience of leaving without knowing how to manage the food allergy.

“We weren’t given any information or told: ‘maybe this is what it is, if he is allergic, these are maybe some of the precautions or this is what you need to do from here’”.

Only 2 participants reported not receiving information from their family physician; most information was received after the allergist visit. All participants searched online for food allergy information. They were more likely to simply “Google” the topic rather than seek specific allergy and anaphylaxis sites, unless directed by health professionals or friends. Participants expressed the need for better education and its dissemination (i.e., reading material, referral to support groups or follow-up with a nurse), as many experienced difficulty absorbing all the information at their first encounter with a health care professional.

#### Theme 3: family anxiety and lifestyle changes

Nearly all participants (94%) experienced anxiety after their child’s first reaction:

“The frightening part was what could have been. And so that was a shock to the system, that I had nightmares for weeks on end after that…”

In response to the anxiety they felt, participants made lifestyle changes socially (88%) or at school (35%). A recurrent theme was significant anxiety regarding family gatherings and other family members feeding their food-allergic child.

“Just to relieve my own anxiety, I’ve turned into a control freak and I’ve had to have all the family gatherings at my house so at least I know that everything is safe.”

Other lifestyle changes were the exclusion of their children from birthday parties and restaurants. Eight participants reported significant difficulties eating outside of the home. In the school and daycare environment, three parents felt comforted with the strict “no-food sharing” policies at their daycares but many worried about kindergarten and beyond where there is less supervision during meals.

## Discussion

Anxiety resulting from the experience of a first food allergic reaction was the predominant theme identified in this study. Other important findings were the lack of provision of information and education about food allergy and anaphylaxis management, and lengthy wait times for referral to allergists. Allergists were the most common source for educational resources on food allergy, anaphylaxis, and epinephrine auto-injectors. This is significant since patients wait for months to see an allergist, and have little information and education in the interim. This is an area on which to focus to ensure that primary care and first line health care workers are educated, and dispense appropriate material to patients and families.

The study also identified a large proportion of individuals who were not receiving information on proper management of their child’s condition. Despite a certain level of confidence in managing food allergic reactions (as reflected by an average score of 2.5 on a 4 point scale of confidence in these topics in the online survey), a significant level of anxiety still exists amongst parents and caregivers. One reason may be the nature of the condition, where parents must initiate lifesaving treatment prior to receiving medical attention. In the study by Gupta et al., they similarly showed good parent baseline knowledge on food allergy but also demonstrated many misconceptions about the topic [25]. The other important finding is that parents and caregivers preferred education and information in the form of written and online materials. These resources are already available from patient websites such as Anaphylaxis Canada (http://www.anaphylaxis.ca), and Anaphylaxis Campaign (UK) (http://www.anaphylaxis.org.uk), and through information packages from Health Canada (http://www.hc-sc.gc.ca), Allergy/Asthma Information Association (http://www.aaia.ca), and the Food Allergy Research & Education (http://www.foodallergy.org). Caregiver knowledge gaps may also be related to the lack of awareness of front-line health care workers regarding available resources.

**Figure 1:**
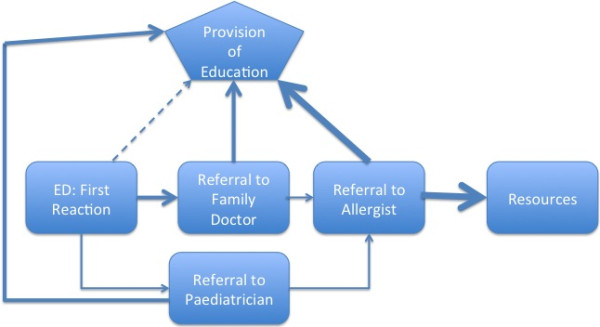
Provision of education as indicated by study participants.

**Figure 2:**
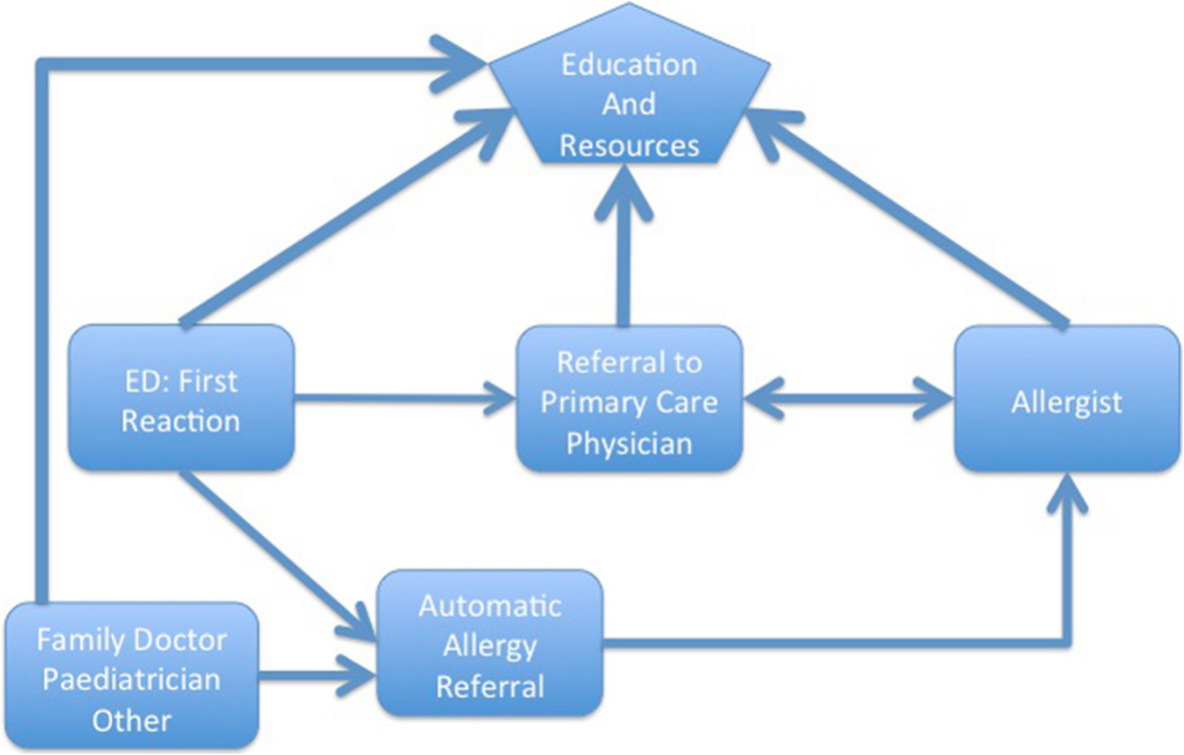
Proposed model for disseminating education.

Figure 1 shows the model for how survey participants were provided education after a first allergic reaction. The model reflects the delay in education and resources as identified by participants, with the heavier weighted arrows indicating where most patients receive information and education. We propose an alternate model (Figure 2), which shows the provision of education to patients and their caregivers earlier in the process, which may facilitate more effective dissemination of information. These models depict the continuum of care and provision of education, incorporating the information from various time points (elucidated from both the quantitative and qualitative data) from first reaction to diagnosis.

To our knowledge, this is the first mixed-methods study to investigate parent and caregiver perspectives on their child’s first food allergic reaction, and to examine what information and resources they were given and at what point in their care this occurred. Previous studies have examined the individual patient experiences [26], and many have investigated the quality of life of patients and their families experiencing allergic reactions and anaphylaxis [27-29]. Other studies have assessed the information needs of families, thus identifying similar information gaps (i.e. anaphylaxis management) [17,20], and the need for educational resources for patients and their families [30,31]. With the use of a mixed-methods design, we are able to confirm our survey findings with those from the qualitative interviews, which allowed us to generate a continuum of care, incorporating multiple healthcare interactions at various time points. The data also shows the time points in the continuum of care where patient and family experience of the first episode of allergic reactions may be improved (i.e. at the first health care encounter). Finally, results identify the need for the shift of the education and resource provision from being an end point (i.e. after allergist assessment) to a continuous delivery of care (i.e. at every contact in the continuum).

There are a number of limitations with this study. Firstly, the survey participants were primarily from Ontario, representing a potential selection bias—approximately 40% of the Canadian population resides within Ontario compared with 71% of our participants. This likely reflects the composition of the Anaphylaxis Canada membership database, which composed our sample population. To counteract this potential bias, we recruited an equal proportion of participants from across Canada to participate in the qualitative telephone interviews. The fact that membership of the Anaphylaxis Canada mailing list is voluntary basis may have also biased our sample as these families were likely more motivated. Another limitation is that the diagnosis is based on self-report and did not require any medical confirmation. In addition, the phase one survey did not require an answer for each question, resulting in some missing data. However, the average response rate was 79% for each question. The survey was also Internet-based and only accessible to those with Internet access. Another potential source of bias is the fact that participants were not excluded if they had prior experience with food allergy personally or with another child. If there is a significant portion with prior experience then they may be more knowledgeable than the general population. Hence, there may an underestimation in the knowledge gaps. Finally, the major issue of wait times may be a reflection of the single payer health care system used within Canada, so the results may not be generalizable to different health care models in other countries.

Next steps of our work will include using the study findings to 1) inform the development of educational strategies for patients newly diagnosed with food allergy (e.g. placement of resources in primary care offices and urgent care settings, educational programs for primary care physicians and allied health, and promoting online resources); 2) to improve the direct referral of patients from the ED to an allergist and the provision of epinephrine auto injector prescription and information in these settings; and 3) to develop strategies to improve the flow and speed that patients and families are moved through the continuum of care from the first encounter to formal diagnosis by the allergist.

Understandably, emergency rooms cannot have information for every food allergen available but more general documents such as signs and symptoms of anaphylaxis as well as proper use of epinephrine auto injectors can be easily made available. In addition, this information sheet can have websites to refer patients and caregivers for further information including Anaphylaxis Canada (http://www.anaphylaxis.ca), Health Canada (http://www.hc-sc.gc.ca), Allergy/Asthma Information Association (http://www.aaia.ca), and the Food Allergy Research & Education (http://www.foodallergy.org). This would be in keeping with other forms of educational materials distributed at the emergency room. In addition, there can be a hesitation by emergency room physicians to make the direct referral due to the concerns of not keeping the primary care physician in the continuum of care. This can be addressed by including the primary care physicians name within the referral letter as the physician who will be following the patient on discharge. This is currently used on other direct from ER referrals to services such as asthma education at some children’s hospitals in Canada.

## Conclusions

This mixed methods study highlighted the multiple deficiencies that exist at different levels of the health system for caregivers of children with a first food allergic reaction. Qualitative interviews reinforced survey results demonstrating a lack of information on food allergy management, when it is most needed, namely at the start of care (i.e., during ED and family physician visits). Findings will inform the development of educational strategies to address these gaps.

## Competing interests

Dr. Waserman has served on advisory boards and received honoraria from King Pharmaceuticals Canada and Pfizer Canada Inc.

Laurie Harada and Laura Bantock are employed by Anaphylaxis Canada.

Heather Cruickshank was employed by Anaphylaxis Canada at the time of the study.

## Authors’ contributions

ZBA this author has directly participated in the design of the research protocol, the development and execution of the study, as well as the analysis and interpretation of data. In addition, ZBA participated in drafting the manuscript and revised it critically for important intellectual content, and has read and approved the final version of the manuscript submitted for publication. MK this author has directly participated in the conception and design of the research protocol, the development and execution of the study, as well as the analysis and interpretation of data. In addition, MK participated in drafting the manuscript and revised it critically for important intellectual content, and has read and approved the final version of the manuscript submitted for publication. CW this author has directly participated in design and execution of the study, as well as the analysis and interpretation of data. In addition, CW participated in drafting and revising the manuscript for important intellectual content, and has read and approved the final version of the manuscript submitted for publication. LH this author has directly participated in the conception and design of the research protocol, the development and execution of the study. In addition, LH participated in drafting and revising the manuscript for important intellectual content, and has read and approved the final version of the manuscript submitted for publication. LB this author has directly participated in the conception and design of the research protocol, the development and execution of the study. In addition, LB participated in drafting and revising the manuscript for important intellectual content, and has read and approved the final version of the manuscript submitted for publication. HC this author has directly participated in the conception and design of the research protocol, the development and execution of the study. In addition, HC participated in drafting and revising the manuscript for important intellectual content, and has read and approved the final version of the manuscript submitted for publication. SW this author has directly participated in the conception and design of the research protocol, the development and execution of the study, as well as the analysis and interpretation of data. In addition, Dr. SW participated in drafting the manuscript and revised it critically for important intellectual content, and has read and approved the final version of the manuscript submitted for publication.

## Supplementary Material

Additional file 1 Structured Interview Guide for qualitative portion of study. Click here for file
