# Supplementary material for: Experiencing a first food allergic reaction: a survey of parent and caregiver perspectives
Source: Allergy Asthma Clin Immunol. 2013 May 29;9(1):18. doi: 10.1186/1710-1492-9-18 (PMC3671211; doi:10.1186/1710-1492-9-18)
Supplement: Additional file 1 — Structured Interview Guide for qualitative portion of study. [file 1710-1492-9-18-S1.docx]

Hello, Please may I speak to _____________________________?

Hello, my name is _________________________ and I am calling on behalf of Dr. Susan Waserman regarding your participation in a phone interview to better understand your experience with a first episode allergic reaction or anaphylaxis. This interview is a follow-up study to the Survey you kindly completed a few months ago about your child’s experience with a first time allergic reaction to food.

Thank you very much for agreeing to participate in this phone interview. We will try our best not to take up too much of your time. The interview will take approximately 30 to 60 minutes. I will ask you questions about your child’s reaction, the events after this reaction, and your own perspectives on the whole experience. This interview will be audio-recorded, but your identity will be kept confidential and you will not be identified in any of the documents. The purpose of recording the interview is to analyse the information in greater detail for our study. Your feedback about your experience is much appreciated, and will be very helpful for our study. We want to take what we learn from you to develop educational materials that parents and caregivers of children with allergies can use to better manage this disease,.

Before we begin, I would like to go over the consent process with you. This is to ensure that you understand that there are no risks to you for participating in the interview, and that you have the right to stop at any time during the interview without any consequences. The information you provide will be kept confidential, and it will be used for study purposes only. Do you have any questions? We emailed you a consent form, did you receive it? We would greatly appreciate it if you could fax it back to us as soon as possible to: Fax #______________

I will now begin the interview, starting with a few demographic questions. Please feel free to stop me any time to ask questions during the interview.

**Demographic Questions:**

1. How old was you child at the time of the reaction?
2. Has anyone in your family ever had any previous experience with food allergy? (eg. With family, friends, etc)
3. Is there are family history of food allergy?
4. What is your occupation? (eg at home, working, in school, etc)

**Qualitative Questions:**

1. **Please describe your child’s first reaction.** What did you do? Who did you call? How did you respond?
2. **Please describe what happened after your child’s reaction?**
   1. What was the timeline of diagnosis?
      1. *Who was the first contact with a healthcare professional, eg ER, Walk in clinic, family doctor*
      2. *Where, under what circumstances, and why?; eg. EMS/ER, car to clinic, etc.*
      3. *What happened after the first contact?*
      4. *Were you referred to someone else?*
      5. *How well informed do you think the health care professionals were during your visit with them?*
3. **Expectations and Information:**
   1. What did you think you needed at the time of the first contact? (eg information, contacts, etc)
   2. What did you actually receive from the health care professionals you saw? What did you NOT get?
   3. Did you use any of the information given to you by health care professionals? If yes, how did you use this information?
   4. What would you have rather received from health care professionals? In what format would you like this information to be delivered? Eg booklet, DVD, website
   5. Were you uncertain about anything after visits with a health care professional?
   6. Did you seek information elsewhere? If yes, where? Eg pharmacist, internet, friends
4. **How did you feel at the time of diagnosis?**

Probe *(trying to determine if anxiety was present at the time of diagnosis and if it still exists):*

- 1. At what point leading up to your child’s diagnosis did you feel anxiety and why? *(if respondent talks about anxiety)*
  2. How do you feel now? *(Question getting at whether anxiety has resolved or not)*
  3. *How are you coping on a daily basis since your child had the reaction?*
  4. *What barriers have you encountered since your child’s reaction? (eg work, economic barriers such as food costs, epipen costs, etc)*

1. Is there anything else you would like to add?
2. Do you have any questions?

**Closing**

Thank you very much for participating in this interview. As an appreciation for your time, we would like to give you a gift certificate for $25, either to Chapters/Indigo or Tim Hortons. Which would you prefer? How would you like us to send this to you?
